# Real-time ex vivo monitoring of NK cell migration toward obesity-associated oesophageal adenocarcinoma following modulation of CX3CR1

**DOI:** 10.1038/s41598-024-54390-5

**Published:** 2024-02-18

**Authors:** Eimear Mylod, Fiona O’Connell, Noel E. Donlon, Maria Davern, Caroline Marion, Christine Butler, John V. Reynolds, Joanne Lysaght, Melissa J. Conroy

**Affiliations:** 1 Cancer Immunology Research Group, Department of Anatomy, School of Medicine, Trinity Biomedical Sciences Institute and Trinity St. James′s Cancer Institute, Trinity College Dublin, Dublin, Ireland; 2 Cancer Immunology and Immunotherapy Group, Department of Surgery, School of Medicine, Trinity Translational Medicine Institute and Trinity St. James’s Cancer Institute, St. James’s Hospital, Trinity College Dublin, Dublin, Ireland; 3 Department of Surgery, School of Medicine, Trinity Translational Medicine Institute and Trinity St. James’s Cancer Institute, Trinity College Dublin, Dublin, Ireland

**Keywords:** Chemokines, Innate immune cells, Tumour immunology, Translational immunology, Cancer

## Abstract

Oesophagogastric adenocarcinomas (OAC) are poor prognosis, obesity-associated cancers which may benefit from natural killer (NK) cell-based immunotherapies. Cellular immunotherapies encounter two key challenges to their success in OAC, namely recruitment to extratumoural tissues such as the omentum at the expense of the tumour and an immunosuppressive tumour microenvironment (TME) which can hamper NK cell function. Herein, we examined approaches to overcome the detrimental impact of obesity on NK cells and NK cell-based immunotherapies. We have demonstrated that NK cells migrate preferentially to the chemotactic signals of OAC patient-derived omentum over tumour in an ex vivo model of immune cell migration. We have identified CX3CR1 modulation and/or tumour chemokine profile remodelling as approaches to skew NK cell migration towards tumour. We also report targetable immunosuppressive facets of the obese OAC TME which dampen NK cell function, in particular cytotoxic capabilities. These data provide insights into approaches to therapeutically overcome key challenges presented by obesity and will inform superior design of NK cell-based immunotherapies for OAC.

## Introduction

Oesophagogastric adenocarcinomas (OAC) are a group of obesity-associated cancers, underpinned by severe immune dysregulation and inflammation^1,2^. The dismal 5-year survival rates for oesophageal adenocarcinoma and gastric adenocarcinoma of 20% and 32% respectively, are largely due to poor treatment response rates of less than 30%^3–7^. Furthermore, current immunotherapies available for OAC have an efficacy of less than 25% meaning a large proportion of patients do not derive any clinical benefit from them^8,9^. As such, this growing group of cancer patients urgently require new options, and we propose that novel natural killer (NK) cell-based chemokine-targeted immunotherapies could help address this unmet need.

NK cells are innate effector lymphocytes which can elicit potent anti-tumour activity, however their dysfunction in cancer is well reported^10–15^. Poor NK cell infiltration of solid tumours, including OAC, is associated with poorer prognosis and therefore, boosting their migration to OAC tumours with therapeutic intent is a desirable concept^11–15^. Previous work by our group has shown that NK cells actively migrate towards the chemotactic cues of OAC patient-derived visceral adipose tissue (VAT) and that higher levels of visceral adiposity correlate to lower frequencies of intratumoural NK cells in OAC patients^16^. This, coupled with reports of deficits in NK cell cytotoxic capacity and phenotypic impairments in people with obesity undoubtedly contributes to compromised anti-tumour immunity^10,17–21^. To therapeutically enhance NK cell responses in OAC, the key challenge of erroneous NK cell migration towards extratumoural tissues of OAC patients must be addressed and circumvented, most notably towards the largest depot of VAT, the omentum^22–24^. If NK cell/NK cell therapy homing to tumour can be effectively augmented in patients, the deleterious effects of the immunosuppressive OAC tumour microenvironment (TME) must also be pre-empted and countered to facilitate immunotherapy-mediated reinvigoration of the anti-tumour immune response^25^. As the number of cancer patients presenting with obesity increases, the disruptive effects of visceral adiposity on anti-tumour immunity and immunotherapy efficacy requires immediate attention and must be considered in the clinical setting^10^. Here, we sought to interrogate the challenges presented by visceral adiposity and the immunosuppressive TME in OAC patients and to identify approaches to limit their impacts on the efficacy of potential NK cell-based immunotherapies.

Here, we report that OAC patients with obesity have significantly lower circulating and intratumoural NK cells. Moreover, we unveil immunosuppressive facets of the OAC soluble TME which are unique to patients with obesity, which dampen the activation receptor expression and cytotoxicity of NK cells and modulate their metabolic profile. Crucially, we have generated a novel ex vivo method of measuring immune cell migration between the tissues of patients with obesity-associated cancer. Using this approach, we have demonstrated that NK cells preferentially migrate to the soluble chemotactic signals of OAC patient-derived omentum over tumour. In addition, we have identified modulation of the fractalkine receptor CX3CR1 with E6130 and/or remodelling of the tumour chemokine profile with MIP-1α and RANTES as potential therapeutic approaches to limit NK cell migration towards omentum and augment NK cell migration towards OAC tumour. These data uncover the dual jeopardy faced by NK cells in OAC patients with obesity, by way of erroneous recruitment to extratumoural sites and/or immunosuppression at the tumour site. This study highlights the importance of considering the visceral adiposity of cancer patients when designing NK cell-based therapies and crucially, provides insights into novel approaches to therapeutically overcome the unique challenges presented by the condition of obesity.

## Results

### NK cell frequencies are significantly diminished in the tumour of OAC patients with obesity and this is paralleled by elevated soluble NKR ligands in the obese soluble TME

**Figure 1:**
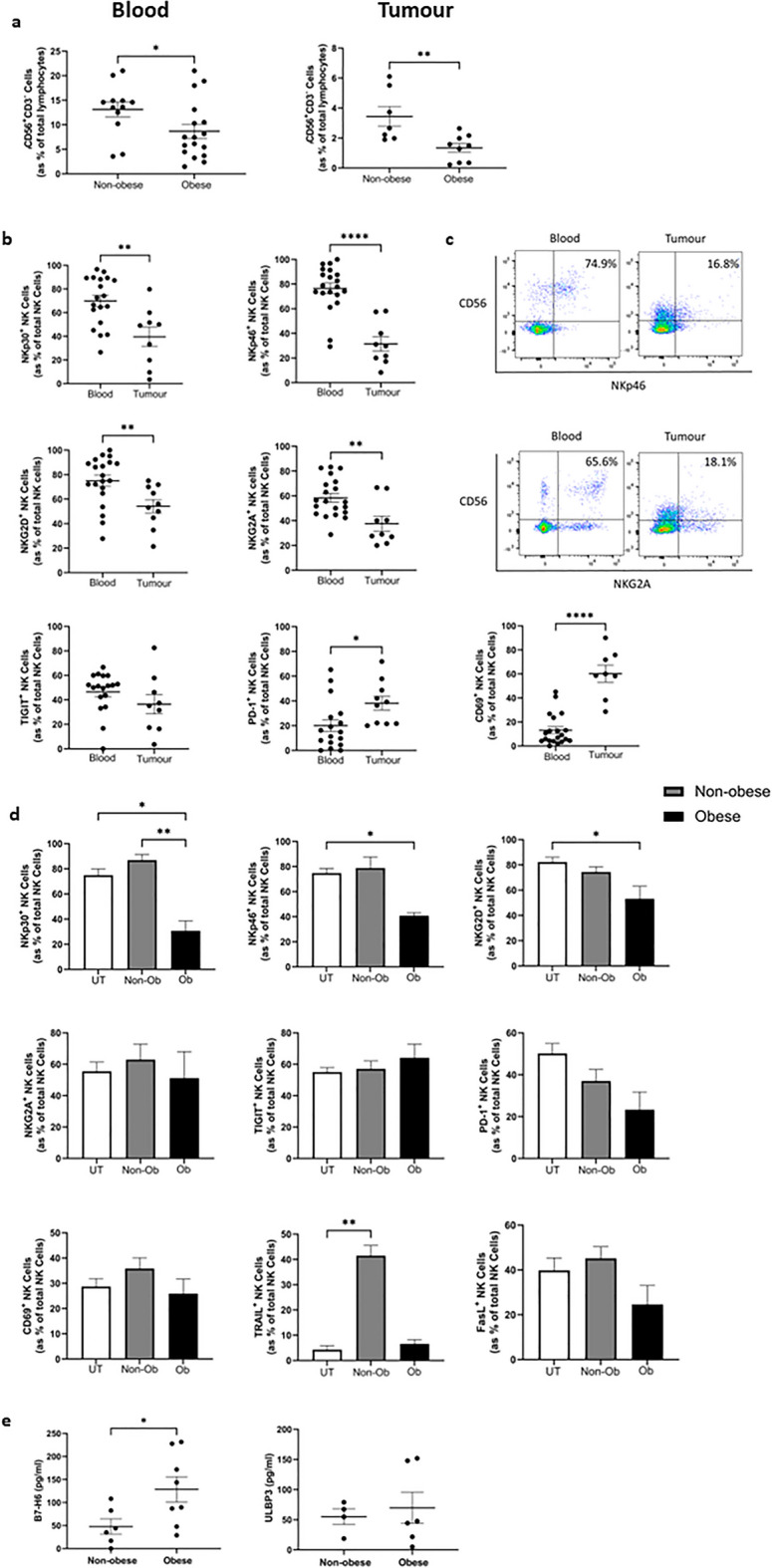
Higher soluble B7-H6 in the TME of obese OAC patients parallels reductions in NKG2D, NKp46 and NKp30 expression on NK cells. (**a**) Dot plots showing the frequencies of CD56^+^CD3^−^ (NK) cells as a percentage of total lymphocytes in the (left) Blood (n = 12, 17) and (right) Tumour (n = 7, 8) of OAC patients categorised into non-obese and obese by visceral fat area. (**b**) Dot plots showing the frequencies of NKp30^+^, NKp46^+^, NKG2D^+^, NKG2A^+^, TIGIT^+^, PD-1^+^ and CD69^+^ NK cells in the blood and tumour of OAC patients. (**c**) Representative dot plots showing the frequencies of NKp46^+^ and NKG2A^+^ NK cells previously gated on the total lymphocyte population in the blood and tumour of OAC patients. Cells shown are gated on total lymphocytes. Percentage frequencies shown are those of the represented populations of positive cells as a percentage of total NK cells. (**d**) Bar charts showing the frequencies of NKp30^+^, NKp46^+^, NKG2D^+^, NKG2A^+^, TIGIT^+^, PD-1^+^, CD69^+^, TRAIL^+^ and FasL^+^ healthy donor NK cells left untreated (white) or following treatment with non-obese (grey) or obese (black) TCM from OAC patients. (**e**) Dot plots showing the levels of B7-H6 and UBLP3 in the TCM of non-obese and obese OAC patients. *p < 0.05, **p < 0.01, ****p < 0.0001 by Mann–Whitney test or Kruskal–Wallis with post-hoc Dunn’s test (Mean ± SEM).

Previous data from our group has indicated a significant negative correlation between visceral obesity and the frequencies of NK cells within OAC patient tumour^16^. In line with these results, here we report significantly lower frequencies of CD56^+^CD3^−^ NK cells in the blood and tumour of OAC patients with visceral obesity (blood; n = 17, tumour; n = 8) as categorised by visceral fat area (VFA) compared to non-obese (blood; n = 12, tumour; n = 7); blood: non-obese versus obese (13.12% vs. 8.6%, *p* = 0.04), tumour: non-obese versus obese (3.466% vs. 1.342%, *p* = 0.0068) (Fig. 1a).

Our group have extensively profiled other immune cells in OAC and unearthed unique phenotypes which exist amongst the circulation, omentum and tumour^22–24,26–30^. However, we have limited knowledge of the NK cell phenotype in the circulation and tumour of OAC patients. There were significantly lower frequencies of activating receptor NKp30^+^, NKp46^+^ and NKG2D^+^ NK cells in the tumour (n = 9–10) of OAC patients compared to the circulation (n = 19–20); NKp30: blood versus tumour (69.92% vs. 39.63%, *p* = 0.0051), NKp46: blood versus tumour (76.58% vs. 31.53, *p* < 0.001), NKG2D; blood versus tumour (75.02% vs. 54.21%) (Fig. 1b,c). Similarly, there were significantly lower frequencies of NKG2A^+^ NK cells in the tumour (n = 9) of OAC patients compared to the circulation (n = 20); blood versus tumour (58.33% vs. 37.6%, *p* = 0.0043) (Fig. 1b,c). Furthermore, there were significantly higher frequencies of PD-1^+^ and CD69^+^ NK cells in the tumour (n = 8–10) of OAC patients relative to the circulation (n = 17–20); PD-1: blood versus tumour (20.03% vs. 38.16%, *p* = 0.011), CD69: blood versus tumour (13.14% vs. 60.18, *p* < 0.0001) (Fig. 1b). There was no significant difference in the frequencies of TIGIT^+^ NK cells between the circulation and tumour of OAC patients (Fig. 1b). These data highlight unique phenotypes in the circulation and tumour of OAC patients and indicate a cytotoxic effector NK cell phenotype exists within the OAC patient circulation which could be redirected to the tumour using chemokine-targeted therapies.

A key issue plaguing the success of cellular immunotherapies is the immunosuppressive OAC TME, which obstructs immunotherapy-mediated reinvigoration of the anti-tumour immune response^25^. As such, we next sought to explore the effects that the soluble microenvironment of the obese (Ob) and non-obese (Non-Ob) OAC patient tumour elicits on healthy donor NK cell phenotype. Treatment of healthy donor NK cells with obese tumour conditioned media (TCM) resulted in significantly lower frequencies of activating receptor expressing NK cells; NKp30: untreated versus obese TCM (74.9% vs. 30.73%, *p* = 0.04), 24 h non-obese TCM versus 24 h obese TCM (86.83% vs. 30.73%, *p* = 0.0066), NKp46: untreated versus 24 h obese TCM (74.87% vs. 40.8%, *p* = 0.02), NKG2D: untreated versus 24 h obese TCM (82.26% vs. 53.2%, *p* = 0.03) (Fig. 1d). Cleavage of activating NK receptor ligands from the surface of cancer cells and their subsequent release into the TME is a key immune evasion strategy employed by solid tumours to impair NK cell-based cytotoxicity^31–33^. As such, we profiled soluble ligands in the TCM and indeed there were significantly higher levels of soluble B7-H6, the ligand for NKp30, in the tumour of obese (n = 8) OAC patients compared to non-obese (n = 6), suggesting that NKR ligand shedding may be exacerbated in obese OAC patients; non-obese versus obese (47.87 pg/ml vs. 128.5 pg/ml, *p* = 0.04) (Fig. 1e). There were no significant differences in the levels of ULBP3 (Fig. 1e). Furthermore, MICA/B were not detected by ELISA and as such, no results are shown. However, exposure to shed soluble B7-H6 has been shown to decrease expression of NKG2D and NKp46 on NK cells, indicating B7-H6 has wide reaching effects beyond its own cognate receptor NKp30^32,34^. TCM from non-obese and obese OAC patients did not alter the frequencies of NK cells which were positive for the inhibitory receptor NKG2A, the immune checkpoints PD-1 and TIGIT or the activation marker CD69 (Fig. 1d).

Surface expression of death receptor ligands FasL and TRAIL were examined before and after culture in obese and non-obese conditioned media to explore the effects of the obese condition on the functional priming of NK cells to elicit killing activity. Previous data from our group has reported elevated levels of these death receptor ligands in the tumour of OAC patients^35^. The frequency of TRAIL^+^ NK cells was significantly higher following treatment with non-obese TCM for 24 h compared to cells left untreated; untreated versus non-obese TCM (4.31% vs. 41.46%, *p* = 0.0096) (Fig. 1d). This parallels previous data from our group which reported significantly lower frequencies of TRAIL^+^ and FasL^+^ NK cells in the circulation of obese compared to non-obese OAC patients, a further indication of the often dysfunctional NK cell phenotype seen in patients with obesity, which can manifest as diminished NK cell cytotoxicity^17,18,35,36^. These data highlight a role for the soluble TME in shaping the phenotype of NK cells in the tumour and highlight the divergent effects of the viscerally obese and non-obese TME in the setting of OAC.

### Obese TCM mediates functional deficiencies in NK cell cytotoxicity and metabolism but promotes a cytokine producing phenotype

**Figure 2:**
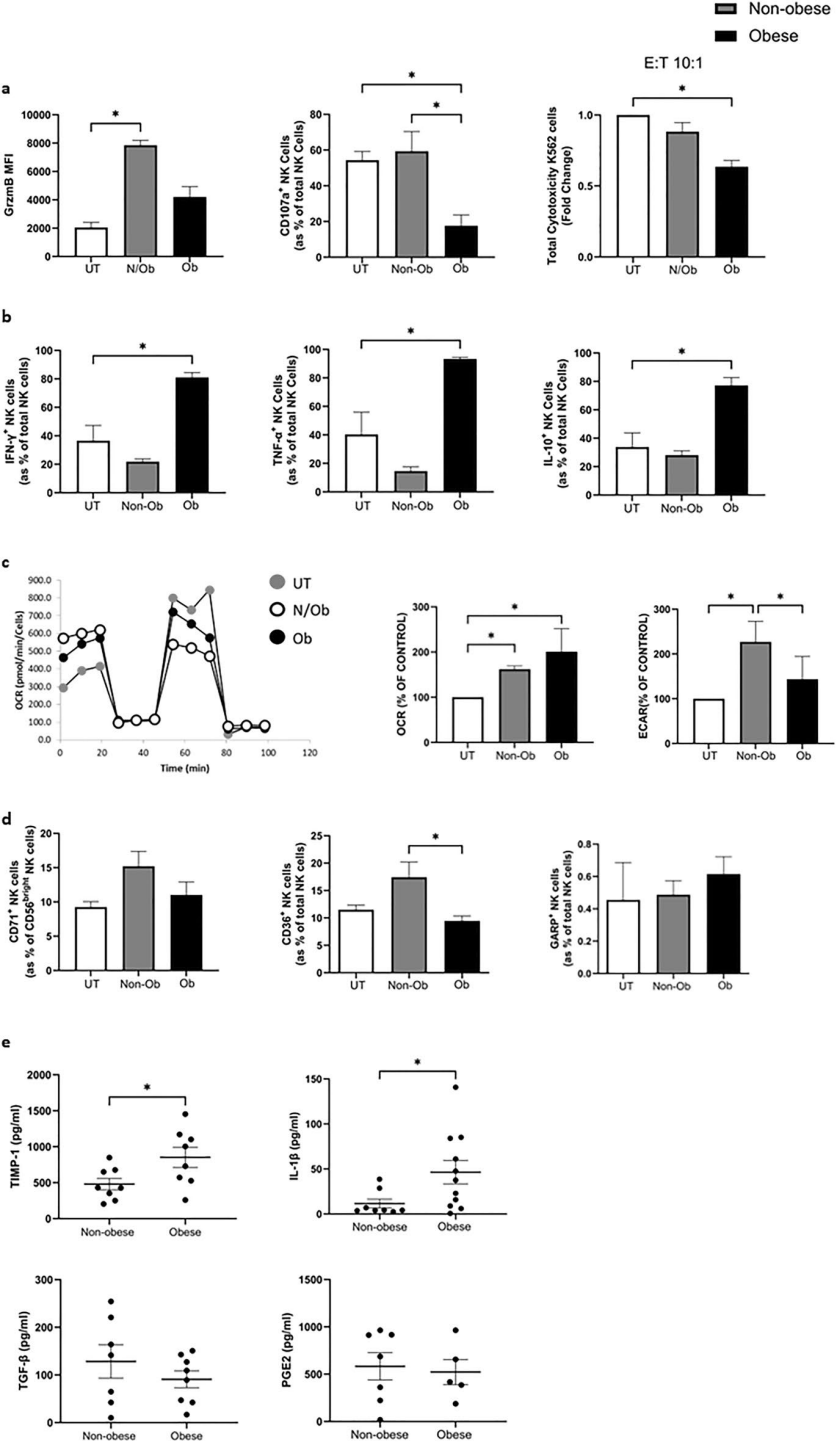
The TME of obese OAC patients dampens NK cell cytotoxic effector function but promotes a cytokine producing profile. (**a**) Bar charts showing the MFI of Granzyme B^+^, frequencies of CD107a^+^ healthy donor NK cells and total cytotoxicity of K562 cells by healthy donor NK cells left untreated (white) or following treatment with non-obese (grey) or obese (black) TCM from OAC patients. (**b**) Bar charts showing the frequencies of IFN-γ^+^, TNF-α^+^ and IL-10^+^ healthy donor NK cells left untreated (white) or following treatment with non-obese (grey) or obese (black) TCM from OAC patients. (**c**) (left) Representative oxidative phosphorylation (OCR) trace of 24 h obese and non-obese TCM treated healthy donor purified NK cells. (right) Bar charts showing the oxygen consumption rate (OCR) and extracellular acidification rate (ECAR) of NK cells treated with non-obese (grey, n = 4) and obese (black, n = 4) TCM for 24 h relative to those left untreated (white, n = 4). (**d**) Bar charts showing the frequencies of CD71^+^, CD36^+^ and GARP^+^ healthy donor NK cells left untreated (white) or following treatment with non-obese (grey) or obese (black) TCM from OAC patients. (**e**) Dot plots showing the levels of TIMP-1, IL-1β, TGF-β and PGE2 in the TCM of obese and non-obese OAC patients. *p < 0.05 by Mann–Whitney test, Kruskal–Wallis test or Freidman test with post-hoc Dunn’s test as appropriate (Mean ± SEM).

Data presented here indicates a compromised NK cell phenotype in the obese TME which may result in diminished capacity to kill tumour cells. We therefore explored the impact of this environment on NK cell killing capacity. The MFI of granzyme B^+^ NK cells was significantly increased when healthy donor-derived NK cells were treated with non-obese TCM but not obese TCM, compared to cells left untreated; untreated versus non-obese TCM (2052 vs. 7852, *p* = 0.014) (Fig. 2a). The frequency of degranulation marker CD107a^+^ NK cells was significantly decreased following treatment with obese TCM compared to cells left untreated or treated with non-obese TCM; untreated versus 24 h obese TCM (54.36% vs. 17.63%, *p* = 0.02), non-obese TCM versus obese TCM (59.36% vs. 17.63%, *p* = 0.03). Finally, NK cell mediated cytotoxicity of K562 target cells was significantly decreased following pre-treatment with obese TCM compared to cells left untreated or pre-treated with non-obese TCM; untreated versus obese TCM, indicating both phenotypic and functional deficits in NK cell cytotoxicity following exposure to obese TCM (Fold change: 1 versus 0.6357, *p* = 0.04) (Fig. 2a).

Data presented here suggests the obese TME may bias NK cells away from a cytotoxic phenotype. As cytokine production is the other central NK cell function, we examined whether there is a shift towards a more cytokine producing phenotype in the obese tumour^37^. The obese but not non-obese tumour environment significantly increased production of both pro- and anti-inflammatory NK cell cytokines. Treatment with TCM from obese OAC patients (n = 3) resulted in significantly higher frequencies of IFN-γ^+^, TNF-α^+^ and IL-10^+^ NK cells compared to those left untreated; IFN-γ: untreated versus 24 h obese TCM (36.6% vs. 81.06%, *p* = 0.04), TNF-α: untreated versus 24 h obese TCM (40.4% vs. 93.4%, *p* = 0.04), IL-10: untreated versus 24 h obese TCM (33.9% vs. 77.2%, *p* = 0.04) (Fig. 2b).

Metabolic changes are integral to NK cell effector functions, including the production of IFN-γ and granzyme B^38,39^. NK cells primarily use two overlapping, glucose-reliant metabolic pathways to generate ATP to fuel their effector functions, mitochondrial oxidative phosphorylation (OXPHOS) and anaerobic glycolysis. NK cells basally utilise OXPHOS and can employ both OXPHOS and glycolysis when activated for extended periods to support their cytotoxic and cytokine producing functions^39,40^. As the TME is often nutrient poor and hypoxic, but abundant in tumour-derived immunosuppressive metabolites which are known to limit NK cell metabolic function, we sought to explore the impact of these soluble environments on NK cell metabolic function^41^.There was a significant increase in the rates of OXPHOS (oxygen consumption rate, OCR) of NK cells treated with both non-obese and obese TCM compared to cells left untreated; untreated versus non-obese (100% vs. 162%, *p* = 0.04), untreated versus obese (100% vs. 292%, *p* = 0.03). However, there was a significant increase in the rate of glycolysis (extracellular acidification rate, ECAR) of NK cells treated with non-obese but not obese TCM, suggesting the non-obese TCM can promote the switch to utilising glycolysis along with OXPHOS when NK cells are activated, whilst obese TCM lacks this effect^39,40^; untreated versus non-obese (100% vs. 227%, *p* = 0.03), non-obese versus obese (227% vs. 143%, *p* = 0.03) (Fig. 2c). There was no significant difference in the frequency of transferrin receptor CD71^+^ CD56^bright^ or GARP^+^ NK cells following treatment with non-obese and obese TCM. There were significantly higher frequencies of lipid scavenger molecule CD36^+^ NK cells following treatment with non-obese TCM compared to cells treated with obese TCM; non-obese TCM versus obese TCM (17.4% vs. 9.4%, *p* = 0.014) (Fig. 2d).

The immunosuppressive TME is characterised by an abundance of immunomodulatory soluble factors including transforming growth factor β (TGF-β) and prostaglandin E2 (PGE2), which have been shown to alter the activating receptor repertoire of NK cells and limit NK cell cytokine production and cytotoxic capacity^42–50^. TGF-β and PGE2 were both present in the soluble OAC TME, however there were no significant differences in the levels of TGF-β or PGE2 in the TCM of obese and non-obese TCM (Fig. 2e). Tissue inhibitors of metalloproteases-1 (TIMP-1) has been shown to counteract TGF-β-mediated immunosuppression of NK cells^51^. There were elevated levels of TIMP-1 and IL-1β in the TCM of obese OAC patients compared to non-obese; TIMP-1: non-obese versus obese (480.5 pg/ml vs. 852.3 pg/ml, *p* = 0.04), IL-1β: non-obese versus obese (11.62 pg/ml vs. 46.47 pg/ml, *p* = 0.03) (Fig. 2e). However, there were no significant differences in the levels of IFN-γ, IL-10, IL-12p70, IL-13, IL-2, IL-4, IL-6, IL-8 or TNF-α in the TCM of obese and non-obese OAC patients (Supplemental Fig. 1A).

### NK cell frequencies are significantly higher in the omentum of OAC patients with obesity and NK cells preferentially migrate towards the soluble cues of OAC patient-derived omentum over that of the tumour

**Figure 3:**
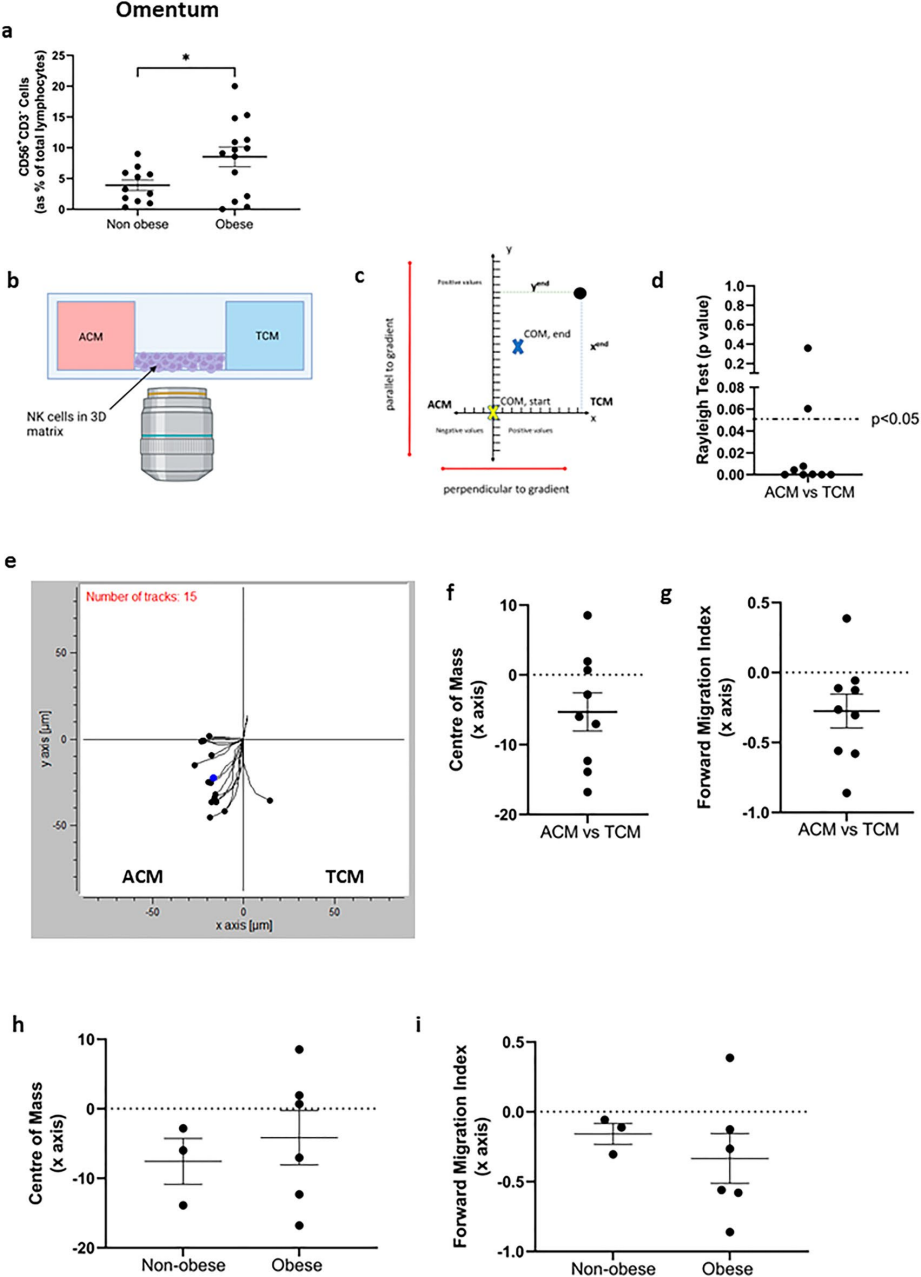
NK cells preferentially migrate towards the soluble cues of OAC patient-derived omentum over tumour, and are significantly more abundant in the omentum of obese patients. (**a**) Dot plot showing the frequencies of CD56^+^CD3^−^ (NK) cells as a percentage of total lymphocytes in the omentum (n = 11, 14) of OAC patients categorised into non-obese and obese by visceral fat area. (**b**) Representative image depicting the set-up of the chemotaxis assay. NK cells were placed in a 3D matrix in the centre and exposed to ACM on the left and TCM on the right. (**c**) Representative plot defining relevant parameters of chemotaxis. The centre of mass (COM) is at point (0,0) at the beginning of an assay. The COM at the end is the average of all cell endpoints (example endpoint indicated by black circle). Along the x axis, NK cells move perpendicular to the gradient. As such, positive values indicate movement towards TCM (positive chemotaxis, right), negative values indicate migration towards ACM (negative chemotaxis, left). (**d**) Dot plot showing the Rayleigh test for 9 independent assays. *p* < 0.05 indicates chemotaxis. (**e**) Representative plot showing transformed migration data along the x and y axis towards ACM on the left and TCM on the right. (**f**) Dot plot showing the centre of mass (COM) along the x axis for 9 independent assays. (**g**) Dot plot showing the forward migration index (FMI) along the x axis, perpendicular to the gradient, for 9 independent assays. (**h**) Dot plot showing the centre of mass (COM) along the x axis of OAC patient derived adipose and tumour conditioned media stratified into non-obese and obese. (**i**) Dot plot showing the forward migration index (FMI) along the x axis, perpendicular to the gradient of OAC patient derived adipose and tumour conditioned media stratified into non-obese and obese. *p < 0.05, **p < 0.01 by Mann–Whitney test (Mean ± SEM). Figures created with Biorender.com.

Previous data from our group has indicated that effector immune cells may be sequestered away to extratumoural tissues in OAC, in particular the omentum^22–24^. This is an issue likely to plague the success of NK cell-based immunotherapies, particularly as the number of patients presenting with obesity increases. Indeed, there were significantly higher frequencies of NK cells in the omentum of OAC patients with obesity (n = 14) compared to their non-obese counterparts (n = 11); non-obese versus obese (3.91% vs. 8.524%, *p* = 0.04) (Fig. 3a).

To examine whether NK cells preferentially migrate towards the soluble cues of OAC patient-derived omentum over tumour, healthy donor-derived NK cells were simultaneously exposed to matched adipose conditioned media (ACM) and tumour conditioned media (TCM) from OAC patients using the Ibidi µslide chemotaxis system (Fig. 3b). There was chemotaxis, as indicated by the Rayleigh test, in 7 of 9 independent assays performed, where matched ACM and TCM from donors were used (Fig. 3d). In this assay, the forward migration index (FMI) of the x axis and the centre of mass (COM) x co-ordinate indicates movement perpendicular to the gradient and as such positive values indicate movement towards TCM and negative values indicate movement towards ACM (Fig. 3c). The COM x axis co-ordinate was negative for 6 of 9 independent assays performed, suggesting NK cells preferentially moved towards the ACM (negative chemotaxis, left) when exposed to both the ACM and TCM (Fig. 3e,f). Similarly, data presented here indicate 8 of 9 independent assays measuring chemotaxis towards matched ACM and TCM had negative FMI values along the x axis and thus indicated that NK cells migrated preferentially towards ACM (Fig. 3e,g). To determine if there was elevated migration towards the ACM of OAC patients with obesity, we categorised patients into obese and non-obese by VFA. However, there was no significant difference in the COM x axis co-ordinate or FMI of the x axis between non-obese or obese OAC patients (Fig. 3h,i). These data indicate NK cells preferentially migrate towards the soluble chemotactic environment of OAC patient-derived omentum over tumour, further highlighting this extratumoural tissue compartment as a potentially significant hindrance to the effectiveness of NK cell-based therapies in OAC.

### Fractalkine is significantly more abundant in OAC patient omentum compared to tumour and modulation of its sole receptor CX3CR1 with E6130 can redirect NK cells towards the soluble cues of OAC patient-derived tumour

**Figure 4:**
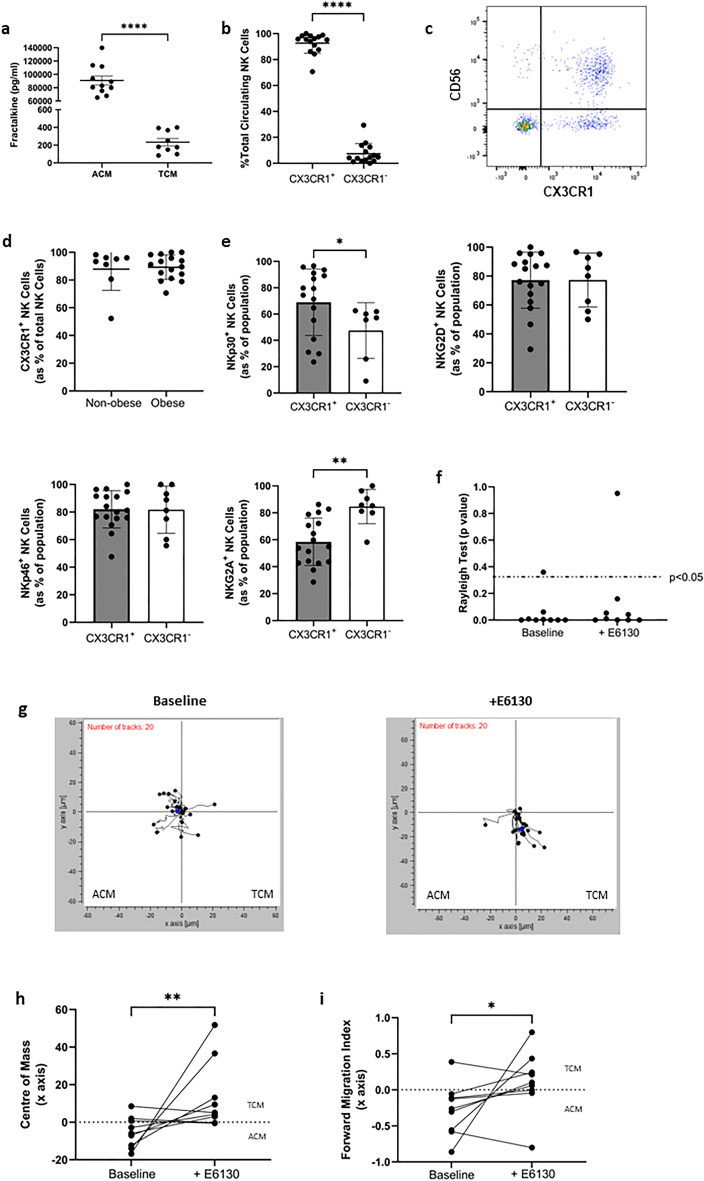
CX3CR1 modulation with E6130 can redirect NK cells away from the soluble cues of OAC patient-derived omentum and towards OAC patient-derived tumour. (**a**) Dot plot showing the levels of fractalkine in matched ACM and TCM of 9 OAC patients. (**b**) Dot plot showing the frequency of CX3CR1^+^ and CX3CR1^−^ NK cell in the blood of OAC patients. (**c**) Representative dot plots showing the frequencies of CX3CR1^+^ and CX3CR1^−^ NK cell previously gated on the total lymphocyte population in the blood of OAC patients. Cells shown are gated on total lymphocytes. (**d**) Dot plot showing the frequencies of CX3CR1^+^ NK cells in the circulation of non-obese and obese OAC patients as categorised by visceral fat area. (**e**) Dot plots showing the frequencies of NKp30^+^, NKG2D^+^, NKp46^+^ and NKG2A^+^ NK cells within the CX3CR1^+^ and CX3CR1^−^ NK cell population in the circulation of OAC patients. (**f**) Dot plot showing the Rayleigh test for 9 independent assays. *p* < 0.05 indicates chemotaxis. (**g**) Representative plots showing transformed migration data along the x and y axis towards ACM on the left and TCM on the right. (**h**) Dot plot showing the COM along the x axis for 9 independent assays. (**i**) Dot plot showing the forward migration index (FMI) along the x axis, perpendicular to the gradient for 9 independent assays. *p < 0.01, **p < 0.01, ****p < 0.0001 by Mann–Whitney or Wilcoxon test as appropriate (Mean ± SEM).

Our group have previously reported abundant levels of the inflammatory chemokine fractalkine in the omentum of OAC patients and identified antagonising its receptor, CX3CR1, as a means to potentially limit NK cell migration to and dysfunction within the omentum^16,24^. Here, in a matched cohort we confirmed significantly higher levels of soluble fractalkine in the ACM of OAC patients compared to the TCM (n = 9); ACM versus TCM (92220 pg/ml vs. 233.6 pg/ml, *p* < 0.0001) (Fig. 4a). There were no significant differences in the levels of fractalkine in the omentum or tumour of OAC patients with visceral obesity or within the non-obese cohorts (Supplemental Fig. 1B). There were abundant levels of fractalkine responsive CX3CR1^+^ NK cells in the circulation of OAC patients (n = 15), indicative of their susceptibility to CX3CR1 modulation; CX3CR1^+^ versus CX3CR1^−^ (92.59% vs. 7.4%, *p* < 0.0001) (Fig. 4b,c). There were no significant differences in the frequencies of circulating CX3CR1^+^ NK cells between OAC patients with visceral obesity (n = 16) and non-obese patients (n = 8), further highlighting the applicability of this approach for OAC patients with visceral obesity and non-obese OAC patients (Fig. 4d).

There were significantly higher frequencies of activating receptor NKp30^+^ NK cells within the CX3CR1^+^ NK cell population in the circulation of OAC patients, relative to the CX3CR1^−^ population; CX3CR1^+^ versus CX3CR1^−^ (68.95% vs. 47.4%, *p* = 0.04) (Fig. 4e). This contrasts inhibitory receptor expressing NKG2A^+^ NK cell frequencies, which were significantly lower; CX3CR1^+^ versus CX3CR1^−^ (58.49% vs. 84.64%, *p* = 0.0011) (Fig. 4e). These data highlight how antagonising CX3CR1 may serve to limit the loss of highly cytotoxic NK cells in the omentum of OAC patients^16,35^.

We have already reported that CX3CR1 antagonism significantly reduces NK cell migration towards the soluble chemotactic signals of the OAC omentum^16^. Here we examined its utility to redirect NK cell migration away from the chemotactic signals of the OAC omentum and towards tumour. To achieve this, NK cells were pre-treated with CX3CR1 modulator E6130 prior to simultaneous exposure to OAC patient-derived ACM and TCM in the Ibidi µslide chemotaxis system. There was chemotaxis, as indicated by the significant Rayleigh test *p* value, in 7 of 9 independent assays performed for both settings (baseline and + E6130) (Fig. 4f). A negative value for the COM x axis co-ordinate and FMI of the x axis indicated movement towards ACM and a positive value indicates movement towards TCM. There was a significant increase in the COM x axis co-ordinate following pre-treatment of NK cells with CX3CR1 modulator E6130 (+ E6130) compared to cells left untreated (baseline) (n = 9); baseline versus + E6130 (− 5.3 vs. 13, *p* = 0.0040) (Fig. 4g,h). Similarly, there was a significant increase in the FMI of the x axis following pre-treatment with E6130 compared to cells left untreated (baseline), suggesting NK cells migrated towards the TCM following pre-treatment with E6130 (n = 9); Baseline versus + E6130 (− 0.27 vs. 1.1, *p* = 0.0188) (Fig. 4g,i). These data indicate increased chemotaxis towards the TCM following pre-treatment with E6130. Using a novel ex vivo approach to simultaneously expose NK cells to two key competing tissue compartments in obesity-associated cancer patients, we have shown that blocking CX3CR1 limits fractalkine-mediated NK cell migration towards the soluble chemotactic cues of the omentum but not the tumour. These data further highlight the potential of CX3CR1 modulation to therapeutically enhance immune infiltration of tumour and potentially boost NK immunotherapy efficacy in obesity-associated cancers.

### Remodelling the chemokine profile of the OAC TME can redirect NK cell migration away from the soluble cues of OAC patient omentum and towards the tumour

**Figure 5:**
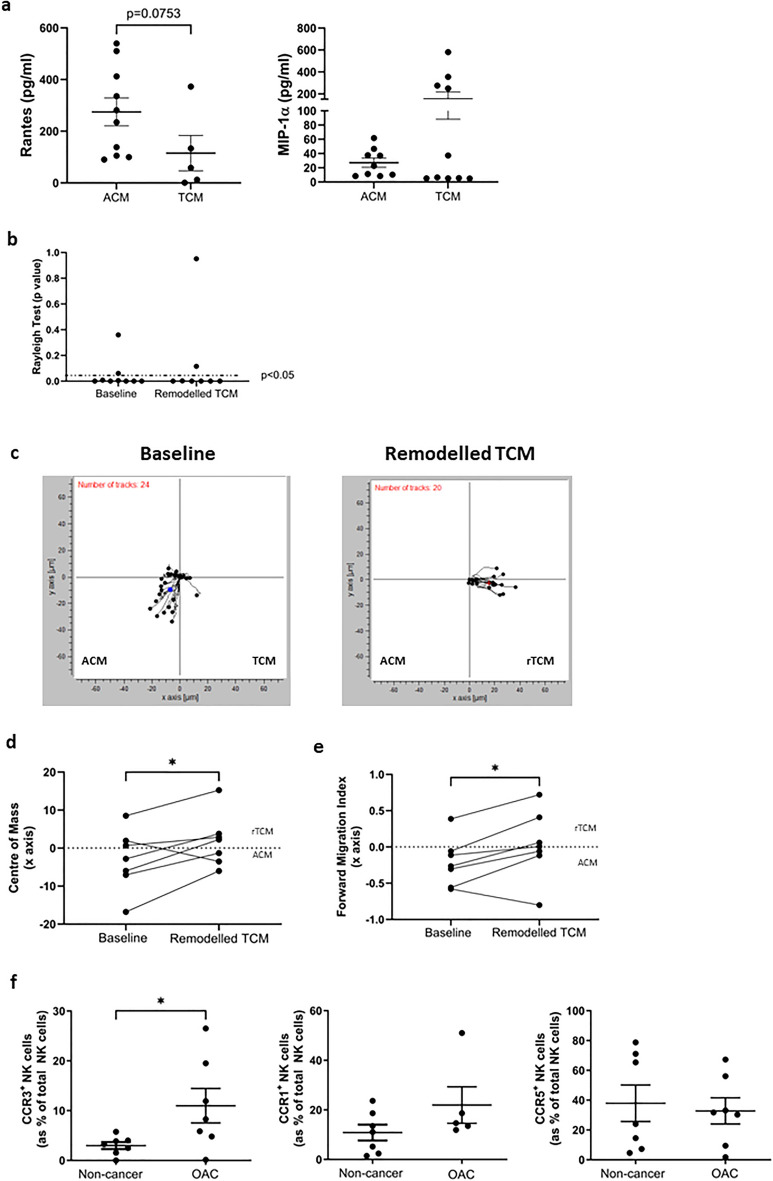
Remodelling the chemokine profile of the OAC TME redirects NK cell migration towards OAC patient-derived TCM and away from matched ACM. (**a**) Dot plots showing the levels of (left) RANTES and (right) MIP-1α in ACM and TCM of OAC patients. (**b**) Dot plot showing the Rayleigh test for 9 independent assays. *p* < 0.05 indicates chemotaxis. (**c**) Representative plots showing transformed migration data along the x and y axis towards ACM on the left and TCM or remodelled TCM (rTCM) on the right. (**d**) Dot plot showing the COM along the x axis for 9 independent assays. (**e**) Dot plot showing the forward migration index (FMI) along the x axis, perpendicular to the gradient for 9 independent assays. (**f**) Dot plots showing the frequencies of (left) CCR3^+^, (centre) CCR1^+^ and (right) CCR5^+^ NK cells in the circulation of non-cancer healthy donor controls and OAC patients. *p < 0.05 by Wilcoxon or Mann–Whitney test as appropriate.

To further interrogate how NK cell migration could be biased towards OAC patient-derived tumour, here the soluble TME was remodelled using recombinant RANTES and MIP-1α (rTCM). Owing to the redundancy of the chemokine system, these chemokines share three key chemokine receptors, CCR1, CCR3 and CCR5, which could be targeted using this approach^52^. As such, we first sought to identify the levels of these chemokines in the ACM and TCM of OAC patients. There were substantially elevated levels of RANTES in the ACM (n = 10) of OAC patients, relative to the TCM (n = 5); ACM versus TCM (274.8 pg/ml vs. 115.4 pg/ml, *p* = 0.07) (Fig. 5a). There were no significant differences in the levels of MIP-1α between the ACM and TCM of OAC patients used in this study. Of note, RANTES was not detected in the TCM of 5 of 10 samples analysed, indicating low to no levels of this chemokine in the TCM. We therefore generated remodelled TCM (rTCM) supplemented with RANTES and MIP-1α as a proof-of-concept to determine whether targeting these chemokine receptor-ligand pairs may be a viable option to increase NK cell migration towards the soluble chemotactic signals of the tumour. There was chemotaxis, as indicated by the Rayleigh test, in 7 of 9 independent assays performed for both settings (Fig. 5b). A negative value for the FMI of the x axis or the COM x axis co-ordinate for these assays indicated movement towards ACM and a positive value indicates movement towards TCM or rTCM (Fig. 5). There was a significant increase in the COM of the x axis when TCM was supplemented with RANTES and MIP-1α (Remodelled TCM) compared to baseline levels (Baseline) (n = 8); Baseline versus Remodelled TCM (− 3.07 vs. 1.89, *p* = 0.0469) (Fig. 5d,e). There was a significant increase in the FMI of the x axis when TCM was supplemented with RANTES and MIP-1α (Remodelled TCM) compared to baseline ACM versus TCM (Baseline), suggesting NK cells migrated towards the TCM when ligands RANTES and MIP-1α were supplemented (n = 8); Baseline versus Remodelled TCM (− 0.21 vs. 0.03, *p* = 0.04) (Fig. 5d,f).

To confirm that remodelling the chemokine profile of the TME would enhance the recruitment of NK cells in the circulation of OAC patients, we sought to confirm expression of these chemokine receptors on the surface of healthy donor and OAC patient NK cells. Indeed, there were significantly higher frequencies of CCR3^+^ NK cells in the circulation of OAC patients relative to non-cancer controls; non-cancer versus OAC (2.983% vs. 11.00%, *p* = 0.02) (Fig. 5f). Furthermore, there were abundant frequencies of CCR1^+^ and CCR5^+^ NK cells in the blood of both OAC patients and healthy donor indicating their availability to respond to a remodelled TME overexpressing these chemokine ligands. These data provide a proof-of-concept that increasing chemokine levels within the tumour can also help boost NK cell migration towards this compartment and highlights tumour chemokine profile remodelling as an approach to alter the immune landscape of OAC tumours to augment tumour eradication^53^.

## Discussion

Obesity-associated cancers such as OAC provide a unique challenge for immunotherapy whereby cytotoxic immune cells are actively recruited to the extratumoural tissues of the omentum and liver^22–24^. We propose such misguided immune cell migration compromises successful NK cell infiltration of the tumour and diminishes anti-tumour immunity, particularly in the OAC patients with the highest levels of visceral obesity, thus posing a significant challenge for immunotherapeutic efficacy in this cohort^16,35^. This is compounded by alterations in NK cell viability, phenotype and function following exposure to the soluble mediators of the OAC omentum, which may compromise their anti-tumour efficacy^23,35^. Furthermore, the solid TME provides a hostile environment for NK cells and NK cell-based therapies^10,54–57^. As such, identifying strategies to improve NK cell/NK cell therapy infiltration of OAC tumour and pre-empting the immunosuppressive barriers within the OAC TME is prudent to effective immunotherapy design, development and efficacy.

Dysfunction of NK cells is well described in obesity, however little is known about the phenotype of NK cells within obesity-associated cancer patients^10,16–19,21,23,35,58–61^. Here, in line with previous data from our group, we report significantly lower frequencies of NK cells in the tumour of OAC patients with visceral obesity, compared to their non-obese counterparts^16^. Higher NK cell frequencies in the obese omentum further highlights this visceral fat depot as a disrupter of anti-tumour immunity in obesity-associated cancers^16,22,23,26,30,35,62^. Targeting the chemokine system to bias immune cell trafficking away from the omentum and towards the solid tumour has potential to invigorate compromised anti-tumour immunity and improve immunotherapy efficacy^10^. However, there is a lack of good models currently available to study obesity-associated cancer and test the applicability of chemokine-based therapies pre-clinically. Here, for the first time, we applied a novel adaption of the ibidi µslide system and OAC patient-derived samples of adipose and tumour tissue conditioned media to simultaneously expose NK cells to the soluble chemotactic cues of what we propose to be two key competing tissue compartments in obesity-associated cancer patients; the omentum and the tumour. Using this approach, we could effectively evaluate the migratory preferences of NK cells and their response to pharmacological interventions. These are the first data to report preferential NK cell migration towards the chemotactic cues of OAC patient omentum over tumour and this is in line with our previous reports of active NK cell migration to OAC patient-derived ACM, further highlighting the threat that the omentum and higher visceral adiposity pose for anti-tumour immunity in OAC^16,63^. We have previously reported compelling evidence that therapeutically precluding NK cell migration to the omentum is warranted in OAC as it would limit omental-induced reductions in NK cell viability and migratory capacity towards tumour, and minimise alterations to NK phenotype and inflammatory profile that perpetuate adipose tissue inflammation^16,63–65^. We have previously confirmed that the chemokine fractalkine is a key driver of erroneous NK cell migration to OAC patient omentum^16^. Here, we have shown that modulating CX3CR1 with E6130 significantly suppresses NK cell migration towards the soluble chemotactic cues from OAC patient omentum and frees them to traffic towards the tumour in an ex vivo model^16,22,23^. Furthermore, there are other rich sources of fractalkine, such as the liver, suggesting that CX3CR1 modulation may serve to limit migration towards these sites^66^. This provides further evidence that CX3CR1-targeted therapies may serve to limit the loss of CX3CR1-expressing cytotoxic effector cells and indeed cellular therapies to the omentum of OAC patients^16,67^. Furthermore, we demonstrate that remodelling the tumour chemokine profile to recruit NK cells is a viable alternative or complimentary approach to help boost NK cell infiltration of OAC tumours to augment tumour eradication^53,68–70^.

Further rationale for therapeutically boosting circulating NK cell migration towards tumour is provided here as we have uncovered the greater availability of key activated subsets of NK cells in the blood of OAC patients compared to tumour. Interestingly, when such circulating activating receptor-expressing NK cells are exposed to the soluble TME of patients with obesity, their surface expression is significantly diminished. These data highlight immunomodulation within the TME as a second key challenge for the successful utilisation of NK cell-based immunotherapies in OAC patients with obesity. Parallels between reduced NKp46, NKp30 and NKG2D and higher levels of B7-H6 within the obese TME suggest that this pathway might be exploited with immunotherapeutic intent via specific matrix metalloprotease (MMP) inhibitors to prevent shedding or ligand neutralising antibodies to neutralise shed ligands within the OAC TME^71^. These data support previous reports from our group which highlighted elevated B7-H6 in the serum and diminished frequencies of NKp30^+^ NK cells in the circulation of OAC patients with visceral obesity^32^. Overall, the obese TME appears to stifle NK cell function, limiting increases in NK cell glycolysis which are required to enhance effector function, in line with other studies in both the obese condition and cancer^21,41,59^. Together, these data support the urgent need to consider the obese condition in immunotherapeutic design and the additional challenges it presents.

Immunotherapies hold great promise in the setting of OAC to reinvigorate the immune infiltrate, however the reasons underlying current response rates of less than 25% require further attention^8,9,72^. Furthering our understanding of the immune landscape of the OAC tumour and indeed extratumoural compartments is central to improving the efficacy of immunotherapies for OAC^72^. Effector lymphocyte migration towards the omentum at the expense of tumour infiltration poses a significant threat to the success of immunotherapies in OAC, other obesity-associated cancers and potentially for all cancer patients who present with the condition of obesity. We highlight CX3CR1 modulation and/or chemokine remodelling of OAC tumour as two promising approaches to redirect NK cell migration away from OAC omentum and towards the tumour. Furthermore, we have identified unique effects of the obese soluble TME on NK cells, which may limit effector function of tumour-infiltrating NK cells and NK cell therapies, and therefore informs the design of future immunotherapies for OAC patients with obesity. Overall, this work unveils novel strategies to address the poor treatment response rates which plague the clinical management of OAC and may offer a pathway to improving immunotherapeutic options and clinical outcomes for patients with this hard-to-treat malignancy.

## Materials and methods

### Patient and healthy donor sample acquisition

The work was performed in accordance with the Code of Ethics of the World Medical Association (Declaration of Helsinki) for experiments involving humans. Patients provided informed consent for sample and data acquisition, and the study received full ethical approval from the St. James’s Hospital Ethics Review Board. Patient samples were pseudonymised to protect the privacy rights of the patients.

A total of 47 patients attending the National Oesophageal and Gastric Centre at St James’s Hospital, Dublin were enrolled in this study from 2017 to 2022. Blood and tumour tissues were collected from 47 patients post-chemoradiotherapy (CRT) at time of surgical resection. Blood sera was collected from 15 patients at time of surgical resection. Body mass index (BMI), visceral fat area (VFA) and anthropometric variables were measured, as described previously (Table 1)^28,73^. The group consisted of 33 males and 12 females, representative of the predominance of OAC in males, with an average age 63.5 years. 21 patients had a diagnosis of oesophageal adenocarcinoma, 18 had a diagnosis of gastro-oesophageal junctional adenocarcinomas and 6 had a diagnosis of gastric adenocarcinoma. The mean BMI at the time of surgery was 28.4 kg/m^2^, making 75% overweight or obese as categorised by BMI. The mean CT-defined VFA was 110.4 cm^2^ making 50% obese by VFA. Neo-adjuvant CRT was administered to 69% of patients with 23 receiving FLOT (5-fluorouracil, oxaliplatin and docetaxel), 7 receiving CROSS (paclitaxel, carboplatin, radiotherapy), 1 receiving FOLFOX (Folinic Acid, 5-fluorouracil and oxaliplatin) and 14 patients receiving no neo-adjuvant CRT (i.e. treatment naïve).

**Table 1:** Patient demographics.

Age (years)	63.5
Sex ratio (M:F)	33:12
Diagnosis (no. patients)	
OAC^a^	21
OGJ^b^	18
Gastric	6
Tumour stage^c^ (no. patients)	
T0	3
T1	10
T2	9
T3	11
T4	9
Nodal status^d^ (no. patients)	
Positive	23
Negative	21
Mean BMI (kg/m^2^)	28.4
BMI (no. patients)^e^	
Underweight (BMI < 19.9)	0
Normal weight (BMI 20–24.9)	10
Overweight (BMI 25–29.9)	19
Obese (BMI > 30)	14
Mean VFA (cm^2^)	110.4
Viscerally obese by VFA^f^	50%
Received neoadjuvant CRT	69%
Treatment received^g^	
FLOT	23
CROSS	7
FOLFOX	1
Naïve	14
Tumour regression grade^h^	
TRG 1–2	6
TRG 3–5	14

^a^Oesophageal adenocarcinoma.

^b^Oesophageal-gastric junctional adenocarcinoma.

^c^Stage unavailable for 3 patients.

^d^Nodal status unavailable for 1 patient.

^e^BMI unavailable for 2 patients.

^f^VFA unavailable for 7 patients, Obese VFA > 160 cm^2^ for men and > 80 cm^2^ for women (Doyle et al.^73^).

^g^FLOT (5-fluorouracil, oxaliplatin and docetaxel), CROSS (paclitaxel, carboplatin, radiotherapy), FOLFOX (Folinic Acid, 5-fluorouracil and oxaliplatin).

^h^Tumour regression grade unavailable for 11 eligible patients.

This study received approval from the Faculty of Health Sciences Research Ethics Committee at Trinity College Dublin, Ireland for provision of healthy, non-cancer control blood. Non-cancer healthy control blood was taken fresh from consenting healthy donors. All data pertaining to non-cancer healthy blood was generated using fresh blood drawn on the day. For NK cell expansion, NK cells were isolated from peripheral blood mononuclear cells prepared from buffy packs obtained from the Irish Blood Transfusion Service in St. James’s Hospital.

### Sample preparation

Blood and tumour biopsies were collected during surgical resection. Tumour biopsies were enzymatically digested with collagenase type IV as previously^16,27^. Adipose tissue conditioned media (ACM) and tumour tissue conditioned media (TCM) were prepared as previously described^16,28^. PBMC were prepared by density gradient centrifugation. Non-cancer healthy control primary NK cells were isolated from PBMC by magnetic cell sorting using human NK cell isolation kit (Stemcell) according to manufacturer’s instructions. NK cells were expanded using MACS media (Miltenyi Biotech) supplemented with human AB serum (Sigma) and IL-2 (Peprotech) as per manufacturer’s instructions.

### Phenotypic analysis of NK cells from OAC patient blood and tumour

Whole blood and intratumoural immune cells were stained with CD56-FITC-Viobright, NKG2A-APC, CCR5-FITC, CCR1-APC, CCR3-PE (Miltenyi Biotec), CD3-APC-Cy7, NKp30-BV421, NKp46-PE-Cy7, NKG2D-PE-Cy5, PD-1-PE-Cy7, TIGIT-PE-Cy5, CD69-BV510 (BioLegend). Red blood cells were lysed using BD Lysing Solution (BD Biosciences) as per manufacturer’s instructions. NK cells were quantified as CD56^+^CD3^−^ cells within the lymphocyte gate. Cells were acquired using the CANTO II (BD Biosciences) flow cytometer and analysed using FlowJo software (Tree Star).

### Phenotypic analysis following treatment with OAC patient ACM and TCM

PBMC were isolated from non-cancer controls by density gradient centrifugation and seeded at a density of 1 × 10^6^ cells/ml RPMI supplemented with 10% FBS and 1% pen/strep. Cells were treated with ACM or TCM from non-obese or obese OAC patients for 2 or 24 h. Cells were stained with CD56-FITC-Viobright, NKG2A-APC (Miltenyi Biotec), CD3-APC-Cy7, CD71-PE-Cy7, CD36-PerCP-Cy5.5, NKp30-BV421, NKp46-PE-Cy7, NKG2D-PE-Cy5, PD-1-PE-Cy7, TIGIT-PE-Cy5, CD69-BV510, TRAIL-APC and FasL-BV421 (BioLegend). NK cells were quantified as CD56^+^CD3^−^ cells within the lymphocyte gate. Cells were acquired using the CANTO II (BD Biosciences) flow cytometer and analysed using FlowJo software (BD Biosciences).

### Cytokine profiling of NK cell following ACM and TCM treatment

PBMC were isolated from non-cancer healthy donor controls by density gradient centrifugation and seeded at a density of 1 × 10^6^ cells/ml RPMI supplemented with 10% FBS and 1% pen/strep. Cells were treated with ACM or TCM from non-obese or obese OAC patients for 2 or 24 h. Cells were subsequently stimulated with 30 ng/ml of IL-12 and 100 ng/ml of IL-15 (Immunotools) for a total of 18 h. After 14 h of stimulation, CD107a-PE-Cy7 (Biolegend) was added for 1 h, followed by addition of 10 µg/ml of Brefeldin A (Sigma) for the remaining three hours. Surface staining for CD56-FITC-Viobright (Miltenyi Biotech) and CD3-APC-Cy7 (Biolegend) was performed. Subsequent intracellular staining for GranzymeB-PE-Cy5, TNF-α-APC, IL-10-BV421 and IFN-γ-BV510 (Biolegend) was performed using FIX&PERM Cell Fixation and Permeabilization Kit (Nordic MUBio). Cells were acquired using the CANTO II (BD Biosciences) flow cytometer and analysed using FlowJo software (BD Biosciences).

### NK cell isolation and expansion

PBMC were isolated from healthy donors by density gradient centrifugation. Natural killer (NK) cells were purified from PBMC by magnetic bead separation using an NK cell isolation kit as per manufacturers protocol (STEMCELL Technologies, Canada). NK cells were expanded in NK MACS Medium (Miltenyi Biotec, Germany), supplemented with 1% of NK MACS Supplement (Miltenyi Biotec, Germany), 5% Human AB serum (Sigma, USA), 500 IU/ml IL-2 (Peprotech, UK). Manufacturers protocols for expansion were used. For chemotaxis experiments, NK cells were resuspended at a concentration of 1 × 10^6^ cells per 50 µl of MACS media. Cells were left untreated or treated with 5 nM of E6130 (Medchem express, USA) for 1 h prior to assay commencement.

### NK cell cytotoxicity assay

Expanded NK cells were seeded at a density of 200,000 cells per 100 µl of MACS media and treated with TCM from non-obese or obese OAC patients for 24 h. NK cell cytotoxicity was quantified with the Cell-mediated Cytotoxicity Assay (ImmunoChemistry Technologies). Briefly, K562 target cells were stained with CFSE. NK cells were co-cultured with stained K562 cells for 4 h at effector target ratio of 10:1. SR-FLICA and 7-AAD were used to quantify apoptotic and dead cells respectively. Cells were acquired using the CANTO II (BD Biosciences) flow cytometer and analysed using FlowJo software (BD Biosciences). Total cytotoxicity was quantified as percentage SR-FLICA^+^ early apoptotic cells, SR-FLICA^+^7-AAD^+^ late apoptotic and 7-AAD^+^ necrotic cells.

### Analysis of soluble mediators in OAC patient tumour conditioned media

Human B7-H6 duo-set ELISA, human ULBP-3 duo-set ELISA (R&D), human MICA/B ELISA, human TGF-β, human Fractalkine, human RANTES, human MIP-1α, human TIMP-1 and human PGE-2 ELISA (Assay Genie) were carried out as per manufacturer’s instructions. MSD multiplex ELISA was carried out per manufacturer’s instructions (MSD).

### NK cell seahorse analysis

Expanded NK cells were seeded at a density of 400,000 cells per 100 ul in a well of a 24-well cell culture XFe24 microplates (Agilent Technologies) pre-coated with poly-d-lysine (Sigma). Cells were treated with obese or non-obese ACM or TCM for 24 h. Prior to the commencement of the assay microplates were centrifuged at 1300 rpm for 3 min to allow for adherence. Media was removed and cells were washed with unbuffered Dulbecco's Modified Eagle's medium (DMEM) supplemented with 10 mM of glucose and 10 mM of sodium pyruvate, (pH 7.4) and incubated for one hour at 37 °C in a CO_2_-free incubator. The oxygen consumption rate (OCR) and extracellular acidification rate (ECAR) were measured using a Seahorse XFe24 Extracellular Flux Analyser (Agilent Technologies).

Measurements were normalised to cell number using the crystal violet assay. Cells were fixed with 1% glutaraldehyde for 15 min. The fixative was removed, and cells were washed with PBS and stained with 0.1% crystal violet in PBS for 30 min. Plates were left to air dry overnight and incubated with 50 μl 1% Triton X-100 in PBS on a plate shaker for 30 min. Absorbance was read at 595 nm on a GloMax microplate reader (Promega).

### Slide and collagen gel preparation

Ibidi µslide chemotaxis slides (“ibidi treat” for 3D chemotaxis assays) and plugs (Ibidi, Germany) were placed in the incubator at 37 °C, 5% CO_2_ overnight prior to the assay commencement.

High concentration JellaGel was used to make collagen gel for cell suspensions (Jellagen, UK). Collagen gel was prepared by mixing 50 µl of high concentration JellaGel solution with 6 µl of JellaGel buffer. This mixture was left at room temperature for 1 h. Following this, 5 µl of JellaGel crosslinker was added to the JellaGel/buffer mixture and mixed by pipetting. This was incubated for 3 min until the solution became stiff and difficult to pipette. Fifty microlitre of cell suspension was added to the collagen mixture and mixed by pipetting. Following preparation of the collagen gel/cell suspension mixture, the cell suspension was added to the chemotaxis slide in the centre chamber, represented by the number 1, in Supplementary Fig. 2A. Plugs were added to ports C,D,E and F (Supplementary Fig. 2A). The chemotaxis plate was placed on the lid of a petri dish and 6 µl of collagen gel/cell suspension was dropped on to the top of port A using a pipette. The plate was titled at an angle and 6 µl of air was pipetted out of port B. This was repeated until the gel flushed through. Plugs were removed from ports C,D,E and F and added to ports A and B (Supplementary Fig. 2A). The plate was placed in a petri dish with wet tissue inside to create a humid atmosphere. This was incubated for 45 min at 37 °C, 5% CO_2._

### Experimental conditions

M199 (Gibco, UK) was used as a negative control and base media for all experiments. M199 was spiked with 20% FBS (Gibco, UK) as a positive control to induce chemotaxis. Matched ACM and TCM from OAC patients at time point of surgical resection were used for these experiments. ACM and TCM were diluted 1 in 4 with M199. To remodel the chemokine profile of the soluble tumour microenvironment, TCM was supplemented with recombinant chemokines to generate remodelled TCM (rTCM). TCM was first diluted 1 in 4 with M199 and then supplemented with 6.5 ng/ml RANTES and 650 pg/ml MIP1-α (Peprotech, UK) to generate rTCM.

To simulate competing chemotactic gradients, different medias were loaded into the two side wells. The experimental set-up used is shown is in Table 2. M199 and M199 + 20% FBS were used as negative and positive controls respectively to ensure chemotaxis was taking place. ACM and TCM were used to model the competing chemotactic gradients of the adipose and tumour environments. ACM and remodelled TCM (rTCM) were used to model the competing chemotactic gradients of the adipose environment and the remodelled tumour environment.

**Table 2:** Experimental set up for chemotaxis assays in Ibidi µslide chemotaxis system.

Cell treatment (centre)	Left chamber (1)	Right chamber (2)
Untreated	M199	M199 + 20% FBS
Untreated	ACM	TCM
Pre-treated with E6130	ACM	TCM
Untreated	ACM	rTCM

### Addition of medias to chemotaxis slides

Following the 45-min incubation of the collagen gel and cells suspension within the chemotaxis slides, medias were pipetted into the respective chambers. Sixty-five microlitres of required media was reverse pipetted into each chamber via port C and E respectively as shown in Supplementary Fig. 2A,B. For addition into the left chamber, plugs were added to ports E and F. Media was reverse pipetted into port C. Plugs were added to ports C and D. This step was repeated for the right chamber via port E (Supplementary Fig. 2A,B).

### Imaging

The Lionheart FX automated microscope (Agilent, USA) was prewarmed to 37 °C, 5% CO_2._ Chemotaxis plates were imaged every 30 s for 1.5 h in brightfield at 4× magnification.

### Experimental analysis

Images were exported as PNG and uploaded to image J (NIH, USA—https://imagej.nih.gov/ij/download.html). Image J plugin Manual Tracking (NIH, USA—https://imagej.nih.gov/ij/plugins/manual-tracking.html) was used to track individual cells. Tracked cells were randomly selected from the centre of the image. A minimum of 15 cells were tracked.

Tracked cell data was imported into the chemotaxis and migration tool (Ibidi, Germany—https://ibidi.com/manual-image-analysis/171-chemotaxis-and-migration-tool.html). Data was initialised, calibrated and transformed and the starting point for all tracked cells was set at (0,0). Chemotaxis plots and calculated values were generated by the chemotaxis and migration tool. The Rayleigh test, centre of mass and forward migration index was calculated as measures of chemotaxis^74^. The directness was calculated as a strong indicator of potential chemotaxis^74^.

- The Rayleigh test is a measure of the uniformity of a circular distribution of points (cell endpoints). When p < 0.05, the null hypothesis (uniformity) is rejected, indicating a chemotaxis effect^74^.
  The centre of mass (COM) indicates the average point of all cell endpoints. It has two co-ordinates, one on the x axis and one of the y axis (Supplemntary Fig. 2C). The COM is a strong indicator of chemotaxis and is dependent on the direction which cells have moved. All COM at the start of an experiment is (0,0). In these experiments, negative values along the x axis indicate movement towards the left, whilst positive values indicate movement towards the right^74^. The COM at the end of an experiment is calculated as below:
  $$\mathbf{M}{\text{end}}= \frac{1}{{\text{n}}}\sum_{{\text{i}}=1}^{{\text{n}}}(\mathbf{X}{\text{i}},{\text{end}}, \mathbf{Y}{\text{i}},{\text{end}})$$
- There are two forward migration indices which reporesent the efficiency of the forward migration of cells and how this relates to both axes (Supplemntary Fig. 2C). These values can be either positive or negative depending on the direction which the cell population has moved. The x axis values indicates movement perpendicular to the gradient. The y axis values indicates movement parallel to the gradient^74^. In these experiments, negative values along the x axis indicate movement towards the left, whilst positive values indicate movement towards the right.
  $$\mathbf{y}\mathbf{F}\mathbf{M}\mathbf{I}=\frac{1}{{\text{n}}}\sum_{{\text{i}}=1}^{{\text{n}}}\frac{\mathbf{Y}{\text{i}},\mathrm{ end}}{\mathbf{D}{\text{i}},\mathrm{ accum}}$$
  $$\mathbf{x}\mathbf{F}\mathbf{M}\mathbf{I}= \frac{1}{{\text{n}}}\sum_{{\text{i}}=1}^{{\text{n}}}\frac{\mathbf{X}{\text{i}},\mathrm{ end}}{\mathbf{D}\mathbf{i},\mathrm{ accum}}$$

### Quantification and statistical analysis

Data were analysed using GraphPad Prism version 9 (GraphPad Prism) and was expressed as mean ± SEM or mean + SEM. Wilcoxon test was used to compare between two groups. Krusall–Wallis with post doc Dunn’s test was used to compared between three or more groups. Statistical significance was determined as *p* < 0.05.

### Supplementary Information

Supplementary Figures.

## Data Availability

The datasets generated during and/or analysed during the current study are available from the corresponding author on reasonable request.
