# Supplementary material for: Real-time ex vivo monitoring of NK cell migration toward obesity-associated oesophageal adenocarcinoma following modulation of CX3CR1
Source: Sci Rep. 2024 Feb 18;14:4017. doi: 10.1038/s41598-024-54390-5 (PMC10874956; doi:10.1038/s41598-024-54390-5)
Supplement: Supplementary file 1 — Supplementary Figures. [file 41598_2024_54390_MOESM1_ESM.docx]

**Supplemental Figures**


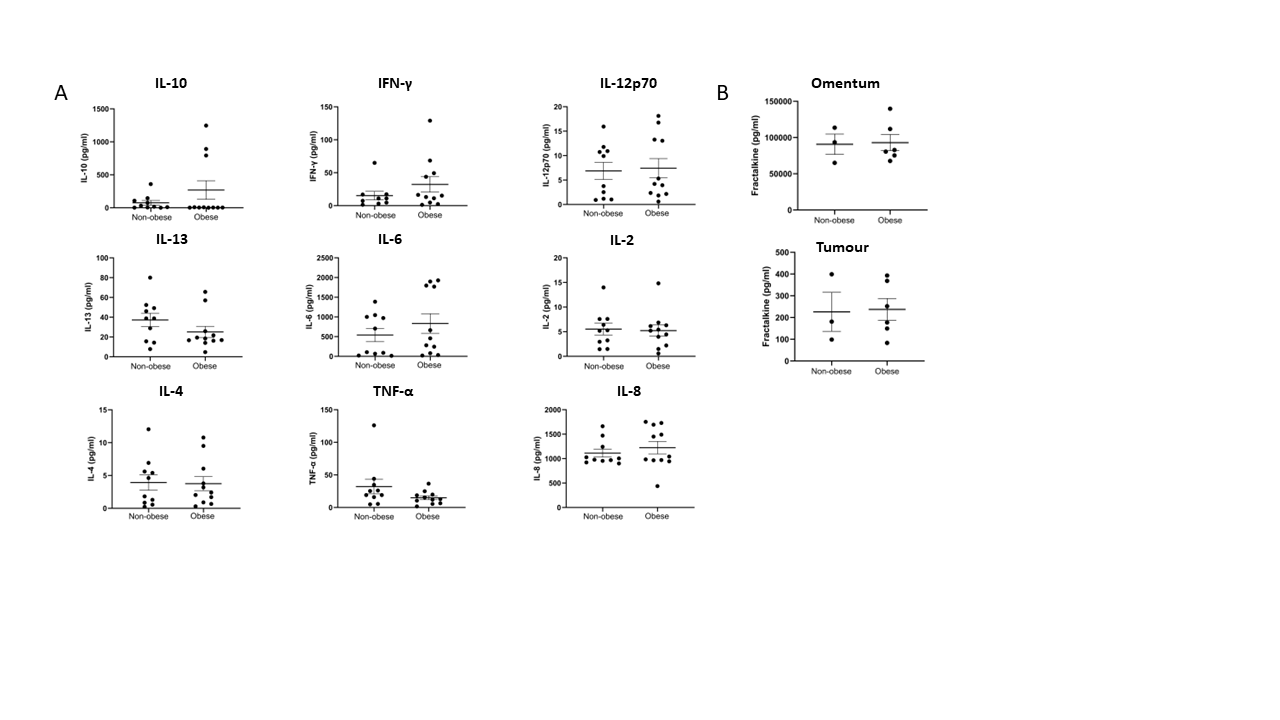


**Supplementary Figure 1: (A)** Dot plots showing the levels of IL-10, IFN-y, IL-12p70, IL-13, IL-6, IL-2, IL-4, TNF-α and IL-8 in the tumour conditioned media of non-obese and obese OAC patients. **(B)** Dot plots showing the levels of fractalkine in the soluble environment of the omentum and tumour of non-obese and obese OAC patients. Wilcoxon test


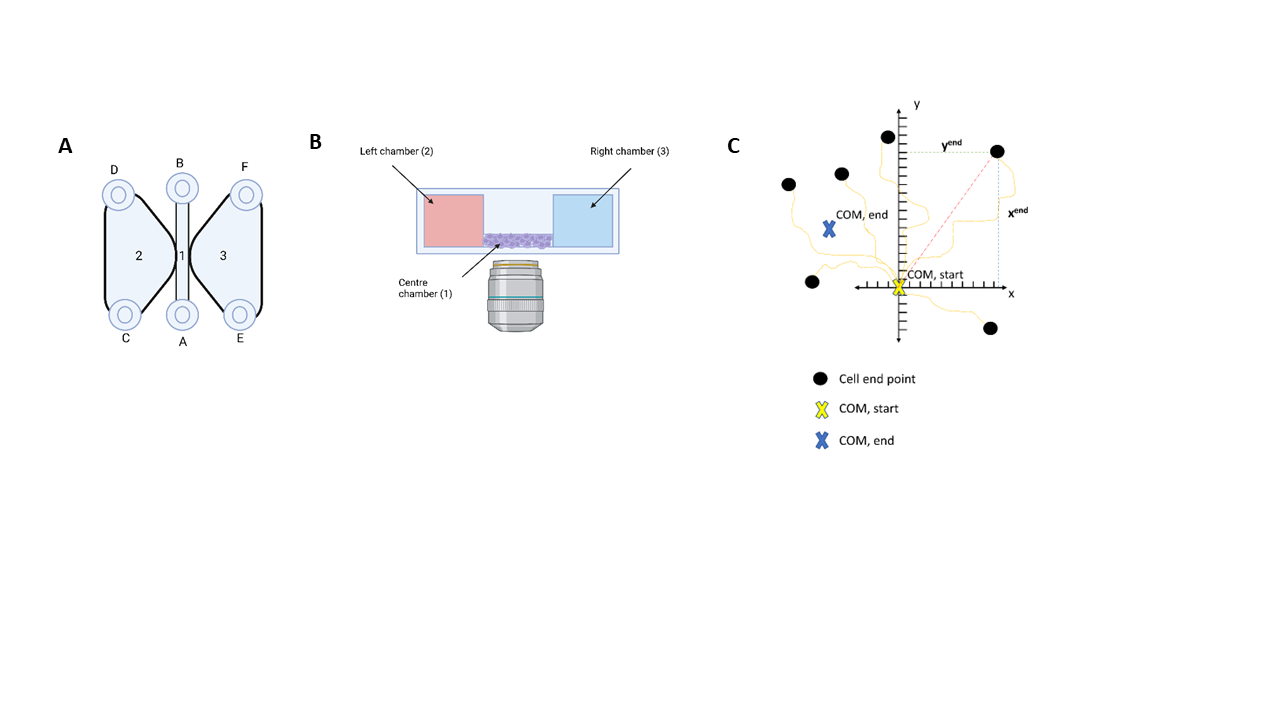


**Supplementary Figure 2: (A) and (B)** µslide set-up**. (C)** Definitions in a 2D trajectory plot. “i” is the index of different cells. Values along the x axis, i.e. perpendicular to the gradient allow for the analysis of directional chemotaxis along the gradient.
